# Trajectories of genetic risk across dimensions of alcohol use behaviors

**DOI:** 10.1111/add.70292

**Published:** 2025-12-23

**Authors:** Jeanne E. Savage, Fazil Aliev, Peter B. Barr, Maia Choi, Gabin Drouard, Megan E. Cooke, Sally I. Kuo, Mallory Stephenson, Sarah J. Brislin, Zoe E. Neale, Spit for Science Working Group, Karen Chartier, Ananda Amstadter, Danielle M. Dick, Emily Lilley, Renolda Gelzinis, Anne Morris, Katie Bountress, Amy E. Adkins, Nathaniel Thomas, Zoe Neale, Kimberly Pedersen, Thomas Bannard, Seung B. Cho, Peter Barr, Holly Byers, Erin C. Berenz, Erin Caraway, James S. Clifford, Megan Cooke, Elizabeth Do, Alexis C. Edwards, Neeru Goyal, Laura M. Hack, Lisa J. Halberstadt, Sage Hawn, Sally Kuo, Emily Lasko, Jennifer Lend, Mackenzie Lind, Elizabeth Long, Alexandra Martelli, Jacquelyn L. Meyers, Kerry Mitchell, Ashlee Moore, Arden Moscati, Aashir Nasim, Jill Opalesky, Cassie Overstreet, A. Christian Pais, Tarah Raldiris, Jessica Salvatore, Jeanne Savage, Rebecca Smith, David Sosnowski, Jinni Su, Chloe Walker, Marcie Walsh, Teresa Willoughby, Madison Woodroof, Jia Yan, Cuie Sun, Brandon Wormley, Brien Riley, Fazil Aliev, Roseann Peterson, Bradley T. Webb, COGA Investigators, Bernice Porjesz, Victor Hesselbrock, Arpana Agrawal, Danielle Dick, Howard J. Edenberg, Tatiana Foroud, Yunlong Liu, Martin H. Plawecki, Samuel Kuperman, Allan Anderson, Jacquelyn Meyers, Laura Bierut, Sarah Hartz, Marc Schuckit, Ronald Hart, Jay Tischfield, Laura Almasy, Alison Goate, Paul Slesinger, Denise Scott, Cathryn Holzhauer, Michie Hesselbrock, Dongbing Lai, John Nurnberger, Leah Wetherill, Xiaoling Xuei, Sean O'Connor, John Kramer, Grace Chan, Chella Kamarajan, Ashwini Pandey, David B. Chorlian, Sivan Kinreich, Gayathri Pandey, Chris Chatzinakos, Jian Zhang, Stacey Saenz deViteri, Christian Richard, Arjun Bingly, Gita Pathak, Andrey Anokhin, Kathleen Bucholz, Fanghong Dong, Alexander Hatoum, Emma Johnson, Vivia McCutcheon, John Rice, Scott Saccone, Zhiping Pang, Sarah Brislin, Jennifer Moore, Alison Merikangas, Miri Gitik, Antti Latvala, Richard J. Rose, Jaakko Kaprio, Danielle M. Dick, Jacquelyn Meyers, Jessica E. Salvatore, Danielle Posthuma

**Affiliations:** ^1^ Department of Complex Trait Genetics, Center for Neurogenomics and Cognitive Research Vrije Universiteit Amsterdam, Amsterdam Neuroscience Amsterdam The Netherlands; ^2^ Department of Psychiatry Robert Wood Johnson Medical School, Rutgers University Piscataway NJ USA; ^3^ Department of Psychiatry and Behavioral Sciences SUNY Downstate Health Sciences University Brooklyn NY USA; ^4^ Veterans Affairs New York Harbor Healthcare System Brooklyn NY USA; ^5^ Department of Psychology, School of Arts and Sciences Rutgers University Piscataway NJ USA; ^6^ Rutgers Addiction Research Center Rutgers University Piscataway NJ USA; ^7^ Institute for Molecular Medicine Finland (FIMM), HiLIFE, University of Helsinki Helsinki Finland; ^8^ Virginia Institute for Psychiatric and Behavioral Genetics, Department of Psychiatry Virginia Commonwealth University Richmond VA USA; ^9^ Institute of Criminology and Legal Policy University of Helsinki Helsinki Finland; ^10^ Department of Psychological and Brain Sciences Indiana University Bloomington IN USA; ^11^ Department of Clinical Genetics Amsterdam UMC, Vrije Universiteit Amsterdam, Amsterdam Neuroscience Amsterdam The Netherlands

**Keywords:** AddHealth, ALSPAC, COGA, Finnish twin cohorts, gene‐by‐development interaction, polygenic scores, Spit for Science, trajectories

## Abstract

**Background and aims:**

Alcohol use behaviors (AUBs) manifest in a variety of normative and problematic ways across the life course, all of which are heritable. Twin studies show that genetic influences on AUBs change across development, but this is usually not considered in research identifying and investigating the genes linked to AUBs. Understanding the dynamics of how genes shape AUBs could point to critical periods in which interventions may be most effective and provide insight into the mechanisms behind AUB‐related genes. In this project, we estimated how genetic influences on AUBs unfold across development using longitudinal modelling of polygenic scores (PGSs).

**Design:**

Using results from genome‐wide association studies (GWASs), we created PGSs to index individual‐level genetic risk for multiple AUB‐related dimensions: *Consumption*, *Problems*, a temporally variable pattern of drinking associated with a preference for beer (*BeerPref*) and externalizing behavior (*EXT*). We created latent growth curve models and tested PGSs as predictors of latent growth factors (intercept, slope, quadratic) underlying trajectories of AUBs.

**Setting:**

PGSs were derived in six longitudinal epidemiological cohorts from the United States, United Kingdom and Finland.

**Participants:**

Participant data were obtained from the longitudinal studies AddHealth, ALSPAC, COGA, FinnTwin12, the older Finnish Twin Cohort and Spit for Science (total *n* = 19 194). These cohorts included individuals aged 14 to 67, with repeated measures collected over a span of 4 to 36 years.

**Measurements:**

Primary measures included monthly frequency of typical alcohol consumption (CON) and heavy episodic drinking (HED).

**Findings:**

When drinking behaviors were averaged across time, higher *Consumption, Problems* and *EXT* PGSs were robustly associated with higher levels of CON and HED (βs ranged from 0.105 to 0.333, *P* < 3.09E‐04) and higher *BeerPref* PGSs with higher HED (β = 0.064, *P* = 3.65E‐05). However, these PGSs were largely not associated with drinking trajectories in the latent growth curve models. In the meta‐analysis, only PGSs for chronic alcohol *Problems* consistently predicted a steeper slope (increasing trajectory) of CON across time (B = 0.470, *P* = 4.20E‐06). Other PGSs were associated with latent growth factors in some individual cohorts, but there was a large degree of heterogeneity.

**Conclusions:**

Genetic associations appear to differ not only between alcohol use behaviors, but also across developmental time points and across cohorts, highlighting the need for genetic studies to take such heterogeneity into account. Individual‐level genetic profiles may be useful to point to personalized intervention timelines, particularly for individuals with high genetic risk scores for alcohol problems.

## INTRODUCTION

Alcohol use behaviors (AUBs) encompass a wide variety of normative and problematic aspects of alcohol consumption, including typical drinking frequency and quantity, heavy episodic (binge) drinking, alcohol‐related problems and clinically significant alcohol use disorders (AUDs). These behaviors are heritable [1, 2, 3], with a complex polygenic architecture influenced by many genetic variants primarily of small individual effect [4, 5]. Genes exert their effects through both broad influences on individual predispositions toward impulsive, rewarding behaviors [‘externalizing’ (EXT)], as well as processes specific to AUBs such as alcohol metabolism [2, 6, 7, 8]. By amassing very large samples, genome‐wide association studies (GWASs) have begun to identify the genes that influence both broad EXT and alcohol‐specific risk [4, 5, 9]. However, these studies usually investigate static AUB measures, neglecting how the associated genetic variants may interact with developmental processes.

AUBs are dynamic phenomena that unfold in different ways across the lifespan, with strong age‐related trends in prevalence as well as substantial variability in these trends [10, 11, 12, 13, 14, 15, 16, 17, 18]. Alcohol addiction is similarly dynamic and thought to involve three distinct stages of neurobiological adaptation that correspond to changes in motivations for drinking and compulsivity of alcohol use [19]. Genetic epidemiology studies in twins have established that the heritability of AUBs changes across the lifetime, with the relative importance of genes increasing until a plateau in middle adulthood [20], followed by a slight drop‐off in older adulthood [21]. Some evidence suggests that there are also qualitative changes in which genes affect AUBs at different life stages [15, 22, 23].

Gene identification studies, however, typically focus on a snapshot of behavior at a single time point, such as a composite measure of current consumption quantity, past‐year alcohol problems or lifetime AUD diagnoses [4, 5, 24]. This approach, while aiming to maximize statistical power, risks missing out on differences critical to the etiology of AUBs such as longitudinal variation or changes in the relative importance of broad versus specific risk processes across time. Existing evidence indicates that the genetic influences on AUBs are not homogeneous, but can be separated into distinct factors related to consumption, problems and patterns of AUBs [25, 26, 27], as well as effects that are shared with EXT or are specific to alcohol use [8]. EXT‐related genes are correlated with early initiation of substance use, whereas alcohol‐specific genes are more relevant for increasing alcohol use/problems over time [8, 28]. The precedence of different neurobiological systems at different stages of the addiction cycle [19] also suggests that different sets of genes may be important for initiation, acceleration and/or persistence of alcohol use. Such different sets of genes have been observed for different stages of smoking behavior and nicotine dependence [4] and are likely to exist for other addictive behaviors.

A recent study [25] used a genomic structural equation modeling approach to identify four genetically distinct factors underlying 18 varied AUBs. These included (1) chronic and severe alcohol ‘Problems’; (2) ‘BeerPref’, a decreasing pattern of alcohol use/problems later in life marked by a preference for drinking beer and drinking without meals; (3) overall quantity and frequency of ‘Consumption’ of varied alcoholic beverage types; and (4) ‘AtypicalPref’, consumption of less common alcoholic beverages such as fortified wine and spirits. Notably, a large proportion of novel genomic loci were identified for the BeerPref factor, which indexed measures such as a reported reduction in drinking over the past 10 years as well as—like the Problems factor—clinically significant consequences of alcohol use. This suggests important genetic distinctions between persistent versus transient measures of alcohol use/problems—distinctions that may not be accurately captured by typical static GWAS phenotypes. However, this interpretation is only speculative because of the cross‐sectional nature of the study. It is as yet unknown whether these genetic factors are prospectively predictive of longitudinal changes in AUBs or how their AUB‐specific effects compare to that of broader EXT risk factors.

Understanding the dynamics of how genes and developmental trajectories interact to shape AUBs could point to critical time points in which interventions would be most effective, as well as improving our understanding of the mechanisms driving statistical genetic associations. In this study, we leverage longitudinal samples to investigate the dynamic effects of genes on AUBs across the lifespan, from early adolescence to middle and older adulthood. Using polygenic scores (PGSs), we examine how the aggregate genetic influences representing EXT‐ and AUB‐related processes shape trajectories of alcohol use across multiple environmental contexts.

## METHODS

This study was pre‐registered under the Open Science Framework (http://osf.io/qg8sa). After registration, two cohorts (described below) were added to the analysis plan as additional suitable datasets were identified after study registration.

### Participants

Data were obtained from six longitudinal studies designed for genetic epidemiology research:

- *n* = 5107 participants from AddHealth, a United States (US) national study of adolescents followed into adulthood [29];
- *n* = 5214 participants from the Avon Longitudinal Study of Parents and Children (ALSPAC), a birth cohort in the United Kingdom (UK) [30, 31, 32];
- *n* = 1955 participants from the prospective subset of the Collaborative Studies on the Genetics of Alcoholism (COGA), a US study of families densely affected with AUDs [33];
- *n* = 1219 participants from Finnish twin cohort (FinnTwin12) and *n* = 6257 from the older Finnish twin cohort (FTC), two twin birth cohorts in Finland [34, 35]; and
- *n* = 4549 participants from Spit for Science (S4S), a cohort of US university students followed throughout college [36].

Data collection included DNA samples for genotyping in addition to interviews or self‐report surveys of behavior and mental health, collected at repeated intervals. In the current study, we use data from participants with ancestry similar to European reference panels (hereafter referred to as ‘EUR’) to match the genetic ancestry background of the discovery GWAS [9, 25]. Full details on data collection for these cohorts have been published previously and are described in the Supplementary Methods. A glossary of commonly used abbreviations is provided for reference in Appendix 1.

### Measures

**FIGURE 1:**
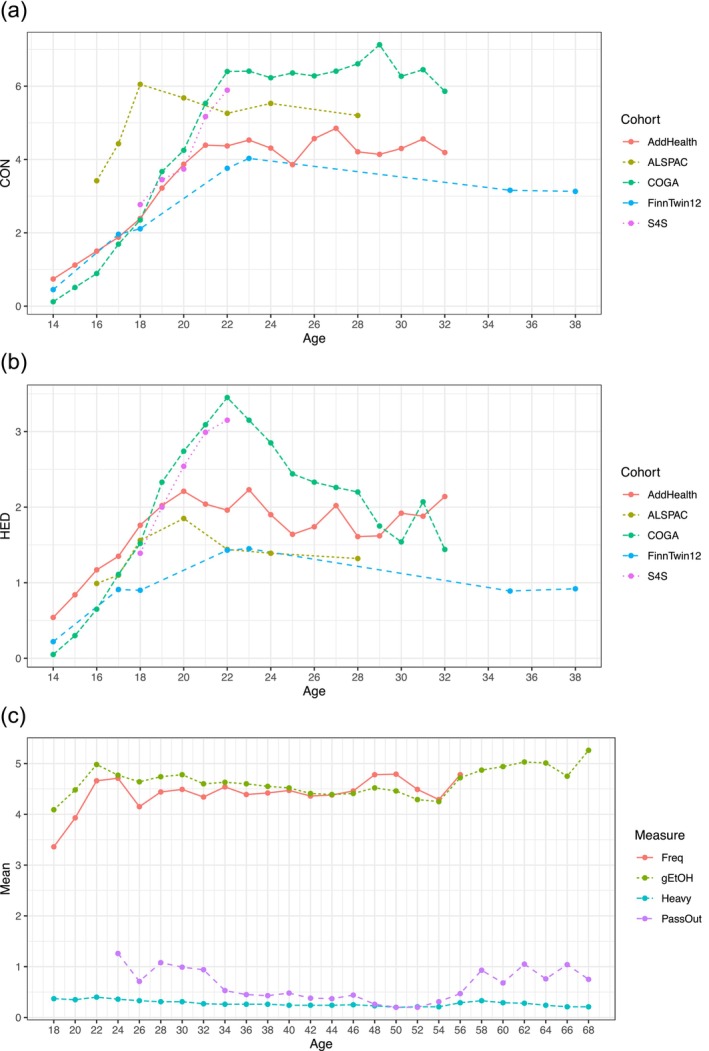
Mean levels per age of (a) typical consumption (CON) and (b) heavy episodic drinking (HED) frequencies in five longitudinal cohorts and (c) multiple alcohol use behaviors in the older FinnTwin cohort.

In each cohort (except FTC), we examined measures of typical frequency of alcohol consumption (CON) and heavy episodic drinking (HED), measured in days per month. Each instance of a repeated measure was recorded along with participants' age at the time of assessment, rounded to the nearest year, with ages ranging from 14 to 38 years old. In FTC, measures included typical frequency of alcohol use (Freq), grams of ethanol consumption per month (gEtOH), a dichotomous indicator of heavy drinking (Heavy) and past year frequency of passing out because of alcohol consumption (PassOut), measured at 18 to 67 years old. Additional details about measure derivations and data cleaning procedures can be found in the Supplementary Methods. A timeline of the assessments for each cohort can be found in Figure 1 and Tables S1–S2.

#### PGSs

PGSs were derived based on GWAS summary statistics of three latent genetic factors described above [25]: Problems, BeerPref and Consumption. This discovery GWAS was carried out in 387 105 EUR individuals from the UK Biobank dataset. A fourth factor, AtypicalPref, was excluded based on prior evidence of its weak genetic signal [25, 37]. To examine broad as well as alcohol‐specific influences, an additional PGS was derived to index EXT based on a prior GWAS [9], which also used a genomic structural equation model approach to aggregate seven impulsivity‐related and substance use phenotypes in a total of 1.5 million EUR samples. The choice of GWAS phenotypes for deriving PGSs was based on their relevance to longitudinal patterns of AUBs, as discussed above, as well as to ensure that there was no overlap with our six study cohorts, which have been included in prior large AUB GWASs (e.g. Saunders *et al*.) [4].

We created these four PGSs in each cohort based on the previously published GWAS summary statistics. Summary statistics were weighted using PRS‐CS (‘auto’ version) and 1000 Genomes EUR reference panels for estimating linkage disequilibrium, as provided with the software [38]. PGSs were calculated in PLINK2 [39] using the ‘‐‐score’ method. Each PGS was entered as a dependent variable in a regression model with the first 10 ancestry principal components and genetic sex as predictors. The standardized PGS residuals, after removing the effects of these covariates, were used for all further analyses to reduce model complexity while accounting for potential confounding effects.

### Data analysis

Data analyses were carried out using R statistical software [40]. First, to examine the overall level of association between the PGSs and AUBs, mean levels of CON and HED were calculated in each sample, averaging across all available waves of data per individual. Each PGS residual was used to predict mean CON and HED in a linear regression model with the *lmer*() or *lm*() functions, for cohorts with (COGA, FinnTwin12 and FTC) or without (AddHealth, ALSPAC and S4S) relatives, respectively. Regression coefficient estimates were meta‐analyzed using random effects inverse variance weighting and restricted maximum likelihood estimation with the *metafor* package [41]. Heterogeneity in the effect size was estimated using the I^2^ statistic, a value from 0% to 100%, which reflects the degree of variability accounted for by between‐study heterogeneity [42].

**FIGURE 2:**
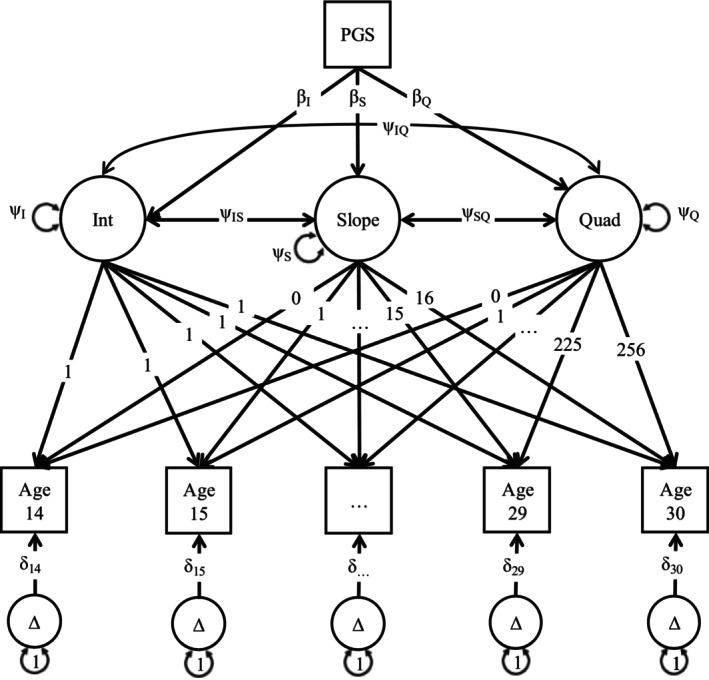
Schematic of the latent growth curve (LGC) model used to estimate the effects of polygenic scores (PGSs) on trajectories of alcohol use behaviors. Measurements of alcohol use are collected at multiple ages, shown in squares. The within‐person covariance between these measures is captured by latent growth factors, shown in circles, which represent the intercept (Int), Slope and quadratic (Quad) parameters that create a line of best fit for an individual's alcohol use trajectory over time. Factor loadings are fixed to 1 for Int, to intervals corresponding with the period between measurements (years) for Slope and to the square of the slope values for Quad. Variance and covariance of the latent growth factors are estimated (Ψ), as are the residual, unexplained variances of the individual measurement occasions (δ/Δ). Of most interest here are the predicted effects (β) of the PGSs on the latent growth factors, which estimate whether an individuals' genetic risk for a particular trait is associated with differences in their values of the latent growth factors and, therefore, point to genetic effects on differential trajectories of alcohol use.

The primary analyses involved latent growth curve (LGC) models, structural equation models that use latent growth factors (intercept, slope and quadratic) to represent the mathematical function underlying the covariance between repeated measures. Here, we fit LGCs for each AUB in each cohort using the *OpenMx* package [43]. First, a baseline model containing either intercept + slope (IS) or intercept + slope + quadratic (ISQ) parameters was fit to determine the general shape of the longitudinal trajectories. After comparing the difference in −2*loglikelihood between models with a χ^2^ test, the best fitting model was selected. Finally, this model was fit sequentially with the addition of each PGS residual (controlling for the same covariates listed above) as an individual‐level predictor of the latent growth factors. This allows us to determine how genetic influences captured by the PGSs shape AUB trajectories (Figure 2). Full information maximum likelihood estimation was used to account for missingness and non‐normality. A multi‐level application (adapted from https://github.com/OpenMx/OpenMx/blob/master/inst/models/nightly/mplus-ex9.12.R) was implemented to account for the non‐independence of observations in samples that included relatives by design (COGA, FinnTwin12 and FTC). Because >90% of participants in each cohort had initiated alcohol use before or during the assessment timeframe, no observations were removed for missing phenotypic values. Individuals were coded with a frequency of zero at each non‐drinking wave.

Models were tested individually in each cohort and meta‐analyzed, as above. The results from FTC were not included in the meta‐analysis because of the difference in age and available measures, but were compared qualitatively with the LGC meta‐analysis results. In FTC, only IS models were selected as many participants only had data for three or fewer measurement occasions, and quadratic effect estimates were unreliable. We applied a study‐wide Bonferroni correction for 56 PGS‐outcome pairs [(5 cohorts * 4 PGSs * 2 outcomes) + (4 PGSs * 4 outcomes in FTC)], resulting in an adjusted α = 0.05/56 = 0.0009.

## RESULTS

Across cohorts, there was a consistent trend of CON and HED increasing sharply from adolescence into emerging adulthood and then leveling off around 22 to 24 years old (Figure 1(a),(b); Table S1). This trend was also apparent in the LGC analyses. Comparing ISQ models, which allow for a quadratic growth factor, to linear IS models showed that the ISQ models provided a better fit in all cases (−2*loglikelihood ratio *P* < 6.34E−08 in all cohorts). Similar longitudinal patterns from 18+ years old were observed in the older FTC sample, although there also appeared to be an uptick in AUBs after 55 years [Figure 1(c)].

All PGSs were robustly associated with higher mean levels of CON and HED when these were averaged across time (Table 1; Figure S1). The only exception was BeerPref PGSs, which were associated with a higher level of HED (meta β = 0.064, *P* = 3.65E−05) but not CON. Consumption PGSs were the strongest predictor of mean CON (meta β = 0.333) while Problems and EXT PGSs were the strongest predictors of mean HED (meta β = 0.154–0.178). In FTC (Table 2), Problems, Consumption and EXT PGSs were significantly associated with higher mean Freq (β = 0.215–0.317), while Problems, BeerPref and EXT PGSs were all associated with higher mean levels of gEtOH, Heavy drinking and PassOut frequency (β = 0.025–0.155). Across all analyses, the within‐sample variance in AUBs accounted for by each PGS was less than 2%.

**TABLE 1:** PGS prediction of mean levels of typical CON and HED frequencies, meta‐analyzed across five longitudinal cohorts.

	AddHealth β	ALSPAC β	COGA β	FinnTwin12 β	S4S β	Meta β	Meta *P*	95% CI	I^2^
Mean CON									
Problems	0.331*	0.286*	0.236	0.156	0.170	**0.237**	**4.01E−10** *	0.163–0.312	54.194
BeerPref	−0.006	−0.282*	−0.135	0.084	0.119	−0.040	0.598	−0.190 to 0.109	88.634
Consumption	0.386*	0.588*	0.443*	0.115	0.146	**0.333**	**3.09E−04** *	0.152−0.513	92.451
EXT	0.171*	0.127	0.050	0.245*	0.309*	**0.195**	**7.94E−07** *	0.118−0.272	57.910
Mean HED									
Problems	0.238*	0.135*	0.259*	0.081	0.117	**0.154**	**2.41E−06** *	0.090−0.218	77.209
BeerPref	0.100	0.037	0.101	0.058	0.117	**0.064**	**3.65E−05** *	0.034−0.095	13.200
Consumption	0.157*	0.106*	0.206	0.053	0.086	**0.105**	**2.16E−06** *	0.062−0.149	51.385
EXT	0.240*	0.140*	0.107	0.158*	0.243*	**0.178**	**1.10E−13** *	0.131−0.225	58.501

*Note*: Columns show the per‐cohort estimates of a linear regression coefficient (β) for the effect of the PGS on mean CON or HED levels, as well as the meta‐analysis of this effect across cohorts (meta β) and its associated *P*‐value and 95% CI. The I^2^ statistic is a metric of between‐cohort heterogeneity in the effect size. Additional abbreviations are found in the glossary (Appendix 1).

Abbreviations: AddHealth cohort, Add Health; ALSPAC, Avon Longitudinal Study of Parents and Children; BeefPref, preference for drinking beer and drinking without meals; COGA, Collaborative Studies on the Genetics of Alcoholism; CON, consumption; EXT, externalizing behaviour; FinnTwin12, FinnTwin12 cohort; FTC, older Finnish twin cohort; HED, heavy episodic drinking; PGS, polygenic score; S4S, Spit for Science.

* Significant after Bonferroni correction for 56 PGS‐outcome pairs, *P* < 0.0009 (bolded values are significant meta‐analysis statistics).

**TABLE 2:** PGS prediction of mean levels of alcohol use behaviors in the older FTC sample.

PGS	β	*P*	95% CI
Freq			
Problems	0.215	3.70E−06*	0.124–0.306
BeerPref	0.118	1.14E−02	0.027–0.210
Consumption	0.317	8.81E−12*	0.226–0.408
EXT	0.250	7.72E−08*	0.159–0.342
gEtOH			
Problems	0.089	1.03E−04*	0.044–0.134
BeerPref	0.105	5.89E−06*	0.060–0.150
Consumption	0.081	4.46E−04*	0.036–0.126
EXT	0.155	1.65E−11*	0.110–0.201
Heavy			
Problems	0.025	4.88E−07*	0.015–0.034
BeerPref	0.034	9.54E−12*	0.024–0.043
Consumption	0.016	1.03E−03	0.007–0.260
EXT	0.046	1.01E−20*	0.036–0.056
PassOut			
Problems	0.086	4.70E−10*	0.059–0.113
BeerPref	0.069	7.50E−07*	0.041–0.096
Consumption	0.016	2.57E−01	−0.012 to 0.043
EXT	0.084	1.10E−09*	0.057–0.112

*Note*: Columns show the estimates of a linear regression coefficient (β) for the effect of the PGS on mean levels of the specified alcohol use behavior and its associated *P*‐value and 95% CI. Additional abbreviations are found in the glossary (Appendix 1).

Abbreviations: BeefPref, preference for drinking beer and drinking without meals; EXT, externalizing behaviour; FTC, older Finnish twin cohort; HED, heavy episodic drinking; PGS, polygenic score; Freq, frequency; gEtOH, grams of ethanol consumption per month; Heavy, heavy drinking; PassOut, past year frequency of passing out because of alcohol consumption.

* Significant after Bonferroni correction for 56 PGS‐outcome pairs, *P* < 0.0009.

In the LGC meta‐analysis (Table 3; Figures S2–S3), Problems PGSs were significantly associated with a higher slope value for CON (meta β = 0.470, *P* = 4.20E−06), meaning that higher genetic risk predicted an accelerated trajectory of increasing drinking frequency over time. There was a general trend that higher PGSs were associated with higher intercept and slope values and lower quadratic factor values (i.e. less leveling off of drinking behaviors over time) for both CON and HED. Some of these were significant in individual cohorts, but these trends were not statistically significant in the combined meta‐analysis. Substantial heterogeneity was observed in the estimates across samples, with I^2^ values of up to 92%. Again, an exception was seen for Problems PGSs predicting CON trajectories, which had not only the strongest effects, but also the most consistent ones (I^2^ = 0%–2%). Given this between‐sample variability, we carried out an exploratory secondary meta‐analysis that (1) excluded the S4S cohort, which had a different measurement time scale than the other cohorts and demonstrated the greatest deviation in estimates; (2) used sample‐size rather than inverse‐variance weighting; and (3) based the effect size on the *t* test statistics of β instead of raw β values, in case measurement scale differences between cohorts were driving the high heterogeneity. In this exploratory analysis (Table S3), heterogeneity remained high. Results were consistent with the primary meta‐analysis, although the effects of EXT PGSs on the CON intercept and Problems PGSs on the HED intercept were now also significant (meta standardized β = 0.276–0.404, *P* < 8.60E−4).

**TABLE 3:** PGS prediction of latent growth factors underlying trajectories of typical CON and HED frequencies, meta‐analyzed across five longitudinal cohorts.

PGS	Factor	AddHealth β	ALSPAC β	COGA β	FinnTwin12 β	S4S β	Meta‐analysis			
							Meta β	Meta *P*	95% CI	I^2^
CON										
Problems	Int	0.036	0.129	0.021	0.013	0.600	0.031	0.064	−0.002 to 0.064	0.43
Problems	Slope	0.462	0.599	0.828	0.290	−2.142	**0.470**	**4.20E−06** *	0.270–0.670	2.05
Problems	Quad	−0.077	−0.343	−0.349	−0.092	2.481	−0.132	0.021	−0.245 to −0.020	0.02
BeerPref	Int	0.028	−0.048	−0.003	0.035	0.269	0.015	0.375	−0.018, 0.049	0.00
BeerPref	Slope	0.159	−0.731	0.429	0.115	−0.617	−0.035	0.877	−0.482 to 0.412	74.25
BeerPref	Quad	−0.206	0.359	−0.424	−0.021	0.678	−0.052	0.707	−0.326 to 0.221	70.43
Consumption	Int	−0.009	0.257*	−0.014	0.004	0.465	0.062	0.318	−0.059 to 0.183	88.55
Consumption	Slope	0.266	1.281*	0.866	0.173	−1.635	0.563	0.034	0.043–1.083	79.22
Consumption	Quad	0.257	−0.709*	−0.142	−0.053	1.874	−0.105	0.596	−0.494, 0.284	84.54
EXT	Int	0.146	0.287*	0.063	0.028	0.587	0.127	0.022	0.018–0.235	85.92
EXT	Slope	0.303	−0.438	0.786	0.857*	−0.828	0.361	0.220	−0.216 to 0.938	83.83
EXT	Quad	−0.229	0.134	−0.714	−0.330*	0.583	−0.268	0.070	−0.559 to 0.022	73.74
HED										
Problems	Int	0.055	0.090*	0.025	0.007	0.432	0.042	0.025	0.005–0.079	64.97
Problems	Slope	0.299	0.095	0.892*	0.134	−1.414	0.285	0.070	−0.023 to 0.593	82.97
Problems	Quad	−0.060	−0.025	−0.486*	−0.023	1.547	−0.103	0.244	−0.277 to 0.071	76.52
BeerPref	Int	0.030	0.085*	0.011	−0.008	0.737	0.029	0.161	−0.012 to 0.070	70.25
BeerPref	Slope	0.155	−0.290	0.592	0.192	−2.404	0.105	0.538	−0.229 to 0.438	85.49
BeerPref	Quad	−0.065	0.241	−0.353	−0.064	2.282	−0.029	0.794	−0.249 to 0.190	85.14
Consumption	Int	0.002	0.008	0.000	−0.005	−0.016	0.000	0.971	−0.020 to 0.019	0.00
Consumption	Slope	0.088	0.519*	0.661	0.104	0.199	0.314	0.022	0.045–0.583	77.67
Consumption	Quad	0.118	−0.374*	−0.333	−0.023	−0.003	−0.140	0.236	−0.373 to 0.092	86.77
EXT	Int	0.141*	0.174*	0.010	0.024	0.576	0.091	0.029	0.009–0.172	92.11
EXT	Slope	0.399*	−0.176	0.699	0.422*	−1.384	0.287	0.110	−0.065 to 0.639	87.05
EXT	Quad	−0.232	0.111	−0.498*	−0.154*	1.381	−0.162	0.157	−0.387 to 0.062	85.99

*Note*: Columns show the per‐cohort estimates of a linear regression coefficient (β) for the effect of the PGS on latent growth factors (Int, slope and Quad) underlying longitudinal trajectories of CON and HED, as well as the meta‐analysis of this effect across cohorts (meta β) and its associated *P*‐value and 95% CI. The I^2^ statistic is a metric of between‐cohort heterogeneity in the effect size. Additional abbreviations are found in the glossary (Appendix 1).

Abbreviations: AddHealth, Add Health cohort; ALSPAC, Avon Longitudinal Study of Parents and Children; BeefPref, preference for drinking beer and drinking without meals; COGA, Collaborative Studies on the Genetics of Alcoholism; CON, consumption; EXT, externalizing behaviour; FinnTwin12, FinnTwin12 cohort; FTC, older Finnish twin cohort; HED, heavy episodic drinking; Int, intercept; PGS, polygenic score; Quad, quadratic; S4S, Spit for Science.

* Significant after Bonferroni correction for 56 PGS‐outcome pairs, *P* < 0.0009 (bolded values are significant meta‐analysis statistics).

In comparison, LGC models within the older FTC cohort (Table 4) demonstrated that BeerPref and EXT PGSs were associated with a higher intercept for Heavy drinking (β = 0.047–0.059, *P* < 1.03E−05). EXT PGSs also predicted higher intercept values for gEtOH (β = 0.197, *P* = 1.12E−06). These differences likely reflect the older age of the sample, as the intercept now references drinking behaviors in young adulthood, after the period of sharp increase observed in adolescence in other cohorts. Again, no significant prediction from the PGSs was seen for the latent growth factors representing longitudinal changes in AUBs.

**TABLE 4:** PGS prediction of latent growth factors underlying trajectories of alcohol use behaviors in middle and older adulthood in the older FTC sample.

PGS	Factor	β	*P*	95% CI
Freq				
Problems	Int	0.218	0.073	−0.020 to 0.456
Problems	Slope	−0.004	0.908	−0.072 to 0.064
BeerPref	Int	0.207	0.092	−0.034 to 0.447
BeerPref	Slope	−0.027	0.446	−0.095 to 0.042
Consumption	Int	0.007	0.956	−0.234 to 0.247
Consumption	Slope	0.087	0.013	0.019–0.155
EXT	Int	0.324	0.009	0.081–0.566
EXT	Slope	−0.020	0.565	−0.089 to 0.049
gEtOH				
Problems	Int	0.091	0.025	0.011–0.171
Problems	Slope	−0.001	0.932	−0.019 to 0.017
BeerPref	Int	0.134	0.001	0.054–0.214
BeerPref	Slope	−0.008	0.412	−0.026 to 0.011
Consumption	Int	0.003	0.937	−0.077 to 0.083
Consumption	Slope	0.019	0.041	0.001–0.037
EXT	Int	0.197	1.12E−06*	0.118–0.277
EXT	Slope	−0.012	0.184	−0.031 to 0.006
Heavy				
Problems	Int	0.025	0.017	0.004–0.046
Problems	Slope	0.000	0.931	−0.005 to 0.004
BeerPref	Int	0.047	1.02E−05*	0.026–0.068
BeerPref	Slope	−0.004	0.117	−0.008 to 0.001
Consumption	Int	0.017	0.112	−0.004 to 0.038
Consumption	Slope	0.000	0.870	−0.005 to 0.004
EXT	Int	0.059	2.14E−08*	0.038–0.080
EXT	Slope	−0.004	0.101	−0.008 to 0.001
PassOut				
Problems	Int	0.250	0.005	0.076–0.424
Problems	Slope	−0.037	0.057	−0.075 to 0.001
BeerPref	Int	0.243	0.008	0.063–0.423
BeerPref	Slope	−0.040	0.049	−0.079 to 0.000
Consumption	Int	−0.118	0.194	−0.297 to 0.060
Consumption	Slope	0.032	0.106	−0.007 to 0.072
EXT	Int	0.172	0.051	−0.001 to 0.344
EXT	Slope	−0.020	0.312	−0.057 to 0.018

*Note*: Columns show the estimates of a linear regression coefficient (β) for the effect of the PGS on latent growth factors (Int, slope and Quad) underlying longitudinal trajectories of CON and HED and its associated *P*‐value and 95% CI. Additional abbreviations are found in the glossary (Appendix 1).

Abbreviations: BeefPref, preference for drinking beer and drinking without meals; EXT, externalizing behaviour; FTC, older Finnish twin cohort; HED, heavy episodic drinking; Int, intercept; PGS, polygenic score; Freq, frequency; gEtOH, grams of ethanol consumption per month; Heavy, heavy drinking; PassOut, past year frequency of passing out because of alcohol consumption; Quad, quadratic.

* Significant after Bonferroni correction for 56 PGS‐outcome pairs, *P* < 0.0009.

## DISCUSSION

Combining multiple international, longitudinal cohorts, we investigated genetic associations with stability and change in alcohol use behaviors across the lifespan. PGSs were used to index multiple AUB‐related dimensions, including EXT, alcohol Problems, alcohol Consumption and a preference for drinking beer and drinking without meals (BeerPref). Despite their robust associations with AUBs on average, most PGSs were not associated with the dynamics of AUB trajectories. However, Problems PGSs significantly predicted a steeper slope of consumption frequency, indicating a genetically influenced acceleration of drinking that is relevant to the development of alcohol problems.

### AUB trajectories

Consistent with previous longitudinal studies [11, 17, 44], we observed strong age‐related trends in prevalence, with AUBs peaking/plateauing in young adulthood. These trends were stable across cohorts from multiple study designs, separated by continents and decades. Genetic associations with these trajectories, on the other hand, were highly variable, largely because of between‐cohort heterogeneity in the effect sizes [45]. This highlights an ongoing challenge in using results from GWASs, in which the hundreds of AUB‐related genes identified through enormous, pooled samples are still only able to account for a small fraction of the known heritability of AUBs [4, 5], likely because of inter‐individual variability in the specific causal genes and processes [45]. Despite focusing on AUB dimensions, defined with a bottom‐up strategy based on their genetic architecture [25], and despite our best efforts to harmonize measures across cohorts, between‐sample heterogeneity remained high in our study.

This likely reflects the variability in sample characteristics of the cohorts included in the current study. As such, meta‐analysis may not be the best approach, because PGSs may index biological processes that are variably relevant for different groups of people or in specific environmental contexts. For example, the Problems PGS seemed to be especially relevant for predicting an increasing, persistent trajectory (Slope and Quad factors) of HED frequency in the high‐risk COGA sample, but not other cohorts. The Consumption PGS, derived from a UK Biobank discovery GWAS, was uniquely associated with latent growth factors underlying CON frequency in the UK‐based ALSPAC sample, which suggests that this factor may reflect predispositions to drinking that are specific to or enhanced by the UK (social) environment. Future research should strive to catalog how the effects of genetic risk and protective factors differ systematically across cohorts and environmental moderators.

### Interpretation of genetic effects

In light of this heterogeneity, it is especially notable that the Problems PGS consistently predicted a steeper slope of consumption frequency—the only significant effect in the LGC meta‐analysis. This is indicative of a robust biological process driving acceleration of alcohol consumption and one that may be specific to the peak in emerging adulthood since the same effect was not observed in the older FTC sample. This difference may, however, be a reflection of the lower heritability of AUBs found in older cohorts [18], likely because of secular changes in social constraints on the expression of genetic predispositions. Although the genes captured by this latent factor include some in common with other AUBs, such as the well‐known alcohol metabolism genes [6], it is only moderately correlated with Consumption, BeerPref or EXT [25]. Further investigation of the genetic influences specific to Problems may, therefore, be helpful to illuminate the causal processes underlying young adult acceleration of alcohol consumption and their links to later alcohol problems.

Results from mean level prediction analyses demonstrated that all four PGSs harbor genetic variants relevant to AUBs, but the growth model results indicated that these may not be the same genes shaping dynamic changes in drinking. Some evidence suggests that, despite the relatively high heritability of AUBs, the genetic influences on trajectories of heavy (episodic) drinking are quite low [17]. If so, this may reflect genetic equifinality, in which high genetic risk leads to high AUB manifestation at some point in life, although the specific timing is shaped by the environment or stochastic effects. For example, if certain genes shape one's susceptibility to drinking in social environments or under stress, these genes will be linked to higher average AUBs when collapsing across a lifetime's worth of social/stressful experiences, but the timing and tempo of these experiences may be driven by the environment or by a wholly distinct set of genes. Other studies, however, have demonstrated that the heritability of latent growth factors for alcohol frequency and quantity is similar to that of cross‐sectional measures of AUBs, approximately 30% to 50% [46, 47]. Further research is needed to resolve this question, and it may be necessary to incorporate longitudinal phenotypes into gene discovery efforts to determine whether the same genes are associated with dynamic versus static AUB measures [48]. The continued low variance in mean AUB levels explained by PGSs suggests that other avenues are worth pursuing in an effort to recapture the ‘missing heritability’, including deep phenotyping [25], longitudinal measures [48] and expanding coverage of the genome to rare and structural variants [49].

### AUB dimensions

By including multi‐dimensional PGSs, we obtained insight into the breadth and specificity of genetic effects. PGSs indexing EXT and Problems were broadly associated with average consumption frequency/quantity as well as heavy (episodic) drinking, while Consumption and BeerPref PGSs were more specifically associated with average frequency or heavy drinking, respectively. BeerPref was even inversely related to consumption frequency in some samples, supporting the hypothesis that this construct represents a genetic predisposition involving a low level of response to alcohol that prompts heavier drinking quantity [37], rather than the social influences that are more relevant to drinking frequency [26]. Our results highlight the need for genetic studies of the diverse heritable risk processes, both broad and specific, which are important for AUBs.

### Limitations and strengths

This study's findings should be viewed in the context of several limitations, including the lack of sample ancestral diversity, small number of AUB measures that could be harmonized across cohorts/waves (lacking important measures like the quantity and types of alcohol consumed, which may be especially relevant to the BeerPref and Consumption factors) and subtle cohort differences in how AUB measures were assessed. We dissect PGS effects that are bound by GWAS discovery power and capture only a small proportion of the variance of AUBs. Nonetheless, these are balanced by strengths of the meta‐analytic approach drawing from complementary cohort study designs, employment of multi‐dimensional PGSs and longitudinal design that explores the changing dynamics of AUBs across the lifespan.

## CONCLUSIONS

We highlight the importance of genetic influences on alcohol Problems in shaping an accelerating trajectory of drinking frequency, which may already be useful for early identification of at‐risk individuals. Multiple other genetic and environmental risk dimensions are, of course, also important in shaping lifelong alcohol use behaviors. Further longitudinal research, especially within gene discovery efforts, is needed to illuminate the mechanisms linking genetic variation to the development of alcohol use behaviors across the lifespan.

## AUTHOR CONTRIBUTIONS

**Jeanne Savage:** Conceptualization (lead); data curation (equal); formal analysis (equal); funding acquisition (equal); visualization (lead); writing—original draft (lead). **Fazil Aliev:** Data curation (equal); formal analysis (equal); writing—review and editing (equal). **Peter Barr:** Data curation (equal); formal analysis (equal); writing—review and editing (equal). **Maia Choi:** Data curation (equal); formal analysis (equal); writing—review and editing (equal). **Gabin Drouard:** Data curation (equal); formal analysis (equal); writing—review and editing (equal). **Megan E. Cooke:** Data curation (equal); writing—review and editing (equal). **Sally I. Kuo:** Data curation (equal); writing—review and editing (equal). **Mallory Stephenson:** Data curation (equal); writing—review and editing (equal). **Sarah J. Brislin:** Data curation (equal); writing—review and editing (equal). **Zoe E. Neale:** Data curation (equal); writing—review and editing (equal). **Spit for Science Working Group:** Data curation (equal); funding acquisition (equal); project administration (equal); resources (equal). **COGA Investigators:** Data curation (equal); funding acquisition (equal); project administration (equal); resources (equal). **Antti Latvala:** Supervision (equal); writing—review and editing (equal). **Richard J. Rose:** Funding acquisition (equal); supervision (equal); writing—review and editing (equal). **Jaakko Kaprio:** Funding acquisition (equal); supervision (equal); writing—review and editing (equal). **Danielle M. Dick:** Funding acquisition (equal); supervision (equal); writing—review and editing (equal). **Jacquelyn L. Meyers:** Funding acquisition (equal); supervision (equal); writing—review and editing (equal). **Jessica Salvatore:** Funding acquisition (equal); supervision (equal); writing—review and editing (equal). **Danielle Posthuma:** Supervision (equal); writing—review and editing (equal).

## DECLARATION OF INTERESTS

D.M.D. is a co‐founder of Thrive Genetics, a member of the advisory board of Seek Health Group, and owns stock in both companies.

## Supporting information


**Table S1.** Descriptive statistics of alcohol use behaviors measured in five longitudinal cohorts.
**Table S2.** Descriptive statistics of alcohol use behaviors measured in the FTC cohort.
**Table S3.** Secondary meta‐analysis of the polygenic score (PGS) prediction of latent growth factors underlying trajectories of typical consumption (CON) and heavy episodic drinking (HED) frequencies.
**Figure S1.** Forest plots of polygenic score (PGS) prediction of mean levels of typical consumption (CON) and heavy episodic drinking (HED) frequencies, meta‐analyzed across five longitudinal cohorts.
**Figure S2.** Forest plots of polygenic score (PGS) prediction of latent growth factors underlying trajectories of typical consumption (CON) frequency, meta‐analyzed across five longitudinal cohorts.
**Figure S3.** Forest plots of polygenic score (PGS) prediction of latent growth factors underlying trajectories of heavy episodic drinking (HED) frequency, meta‐analyzed across five longitudinal cohorts.

## Data Availability

Raw data from this study are available to qualified researchers. AddHealth: dbGaP accession no. phs001367.v1.p1. ALSPAC: https://www.bristol.ac.uk/alspac/researchers/access/. COGA: https://cogastudy.org/. Finnish twin cohort (FinnTwin12 and older cohort): data used in the analysis is deposited in the Biobank of the Finnish Institute for Health and Welfare (https://thl.fi/en/research-and-development/thl-biobank/for-researchers) and is available to researchers after written application and following the relevant Finnish legislation. Spit for Science: dbGaP accession no. phs001754.v4.p2.
