# Supplementary material for: Trajectories of genetic risk across dimensions of alcohol use behaviors
Source: Addiction. 2025 Dec 23;121(5):1153–65. doi: 10.1111/add.70292 (PMC13088926; doi:10.1111/add.70292)
Supplement: Supplementary file 1 — Table S1. Descriptive statistics of alcohol use behaviors measured in five longitudinal cohorts. Table S2. Descriptive statistics of alcohol use behaviors measured in the FTC cohort. Table S3. Secondary meta‐analysis of the polygenic score (PGS) prediction of latent growth factors underlying trajectories of typical consumption (CON) and heavy episodic drinking (HED) frequencies. Figure S1. Forest plots of polygenic score (PGS) prediction of mean levels of typical consumption (CON) and heavy episodic drinking (HED) frequencies, meta‐analyzed across five longitudinal cohorts. Figure S2. Forest plots of polygenic score (PGS) prediction of latent growth factors underlying trajectories of typical consumption (CON) frequency, meta‐analyzed across five longitudinal cohorts. Figure S3. Forest plots of polygenic score (PGS) prediction of latent growth factors underlying trajectories of heavy episodic drinking (HED) frequency, meta‐analyzed across five longitudinal cohorts. [file ADD-121-1153-s001.docx]

*Supplementary Materials for*

**Trajectories of genetic risk across dimensions of alcohol use behaviors**

Jeanne E Savage^1*^, Fazil Aliev^2^, Peter B Barr^3,4^, Maia Choi^5,6^, Gabin Drouard^7^, Megan E Cooke^2^, Sally I Kuo^2^, Mallory Stephenson^8^, Sarah J Brislin^2^, Zoe E Neale^3,4^, Spit for Science Working Group, COGA Investigators, Antti Latvala^9^ Richard J. Rose^10^, Jaakko Kaprio^7^, Danielle M Dick^2,6^, Jacquelyn Meyers^3,4^, Jessica E Salvatore^2^, Danielle Posthuma^1,11^

^1^ Department of Complex Trait Genetics, Center for Neurogenomics and Cognitive Research, Vrije Universiteit Amsterdam, Amsterdam Neuroscience, Amsterdam, The Netherlands

^2^ Department of Psychiatry, Robert Wood Johnson Medical School, Rutgers University, Piscataway, NJ, USA

^3^ Department of Psychiatry and Behavioral Sciences, SUNY Downstate Health Sciences University, Brooklyn, NY, USA

^4^ Veterans Affairs New York Harbor Healthcare System, Brooklyn, NY, USA

^5^ Department of Psychology, School of Arts and Sciences, Rutgers University

^6^ Rutgers Addiction Research Center, Rutgers University, Piscataway, NJ, USA

^7^ Institute for Molecular Medicine Finland (FIMM), HiLIFE, University of Helsinki, Helsinki, Finland

^8^ Virginia Institute for Psychiatric and Behavioral Genetics, Department of Psychiatry, Virginia Commonwealth University, USA

^9^ Institute of Criminology and Legal Policy, University of Helsinki, Finland

^10^ Department of Psychological and Brain Sciences, Indiana University, Bloomington, IN, USA

^11^ Department of Clinical Genetics, Amsterdam UMC, Vrije Universiteit Amsterdam, Amsterdam Neuroscience, Amsterdam, The Netherlands

Table of Contents

[Supplementary Methods 3](#_Toc206066253)

[Participants 3](#_Toc206066254)

[*AddHealth* 3](#_Toc206066255)

[*ALSPAC* 3](#_Toc206066256)

[*COGA* 3](#_Toc206066257)

[*FinnTwin12* 4](#_Toc206066258)

[*FTC* 4](#_Toc206066259)

[*S4S* 4](#_Toc206066260)

[Measures 4](#_Toc206066261)

[*CON* 5](#_Toc206066262)

[*HED* 5](#_Toc206066263)

[*Other AUB measures* 5](#_Toc206066264)

[References 6](#_Toc206066265)

[Appendices 7](#_Toc206066266)

[Supplementary Tables 8](#_Toc206066267)

[Supplementary Figures 11](#_Toc206066268)

# **Supplementary Methods**

## **Participants**

Data were obtained from six existing cohorts, including a national study of adolescents in the US (AddHealth), a birth cohort in the UK (the Avon Longitudinal Study of Parents and Children; ALSPAC), a US study of families densely affected with AUDs (the Collaborative Study on the Genetics of Alcoholism; COGA), two twin birth cohorts in Finland (FinnTwin12 and the older Finnish Twin Cohort [FTC]), and an cohort of university students in the US (Spit for Science; S4S).

### *AddHealth*

AddHealth is an ongoing, nationally representative study of adolescents followed into adulthood in the US. In 1994-1995, participants were selected from a stratified sample of 132 schools, resulting in an initial sample of n=90,118 students in grades 7-12. A subset of the original sample (n=20,745) was selected for additional in-home interviews (Wave I/age 11-18 to Wave 5/age 35-42), with a total of n=15,159 individuals providing samples for genotyping at Wave IV. All participants provided informed consent/assent, and the study was approved by the corresponding university Institutional Review Board. Full details on data collection have been previously published (1). The current study includes n=5,107 unrelated EUR participants.

### *ALSPAC*

Pregnant women resident in Avon, UK with expected dates of delivery between 1st April 1991 and 31st December 1992 were invited to take part in the study, and the initial number of pregnancies enrolled was 14,541, including 13,988 children who were alive at 1 year of age. After additional recruitment efforts, the total sample size for data collected after the age of seven is 15,447 pregnancies, from which 14,901 children were alive at 1 year of age. Study data were collected and managed using REDCap electronic data capture tools hosted at the University of Bristol (2). The study website contains details of all the data that is available through a fully searchable data dictionary and variable search tool (<http://www.bristol.ac.uk/alspac/researchers/our-data/>). Ethical approval for the study was obtained from the ALSPAC Ethics and Law Committee and the Local Research Ethics Committees. Consent for biological samples has been collected in accordance with the Human Tissue Act (2004). Full details on data collection have been previously published (3, 4). The current study includes n=5,214 unrelated EUR participants with available genotypic data and measurements collected at ages 16-28.

### *COGA*

This study, initiated in 1989, recruited high-risk families through adult probands in treatment for alcohol dependence, including probands, their relatives, and community-ascertained comparison families (n=16,848). A prospective study of a subset of adolescents and young adults (aged 12-22) from COGA families was initiated in 2004, with subjects reassessed every two years. Currently, 89% of participants have 2+ interviews completed. All participants and their families provided informed consent/assent, and the study was approved by the corresponding university Institutional Review Board. Full details on data collection have been previously published (5). The current study includes n=1,955 EUR participants from the prospective sample.

### *FinnTwin12*

This population-based study of Finnish twins born 1983–1987 was identified through Finland’s Central Population Registry. A total of 2,705 families (87% of all identified) were enrolled and twins were invited to participate in mailed surveys at ages 12, 14, 17-18, 21-26, and 34-40. An intensively studied subset (1035 families) was selected for genotyping and additional interviews. All participants and their families provided informed consent/assent, including written informed consent for all those who provided blood or saliva samples for DNA genotyping. The study was approved by the Indiana University Institutional Review Board and by the Helsinki University Hospital (HUS) Regional Ethics Committee. Full details on data collection have been previously published (6). The current study includes n=1,219 EUR participants with genetic data passing quality control.

### *FTC*

The older FTC is a population-based study of twins born in Finland before 1958. Four waves of surveys were conducted by mail in 1975, 1981, 1990, and 2011. Ethical approval was obtained from the HUS Regional Ethics Committee (ID 01/2011). Informed consent to participate was not obtained directly but was inferred from the completion of the questionnaires. All those who provided blood or saliva samples for DNA have provided written informed consent. The purpose of the study was explained to the participants, and all participants were aware that they could withdraw from the study at any time without any consequences. A full description of the older FTC has been published previously (7). The current study includes n=6,257 EUR participants with genetic data passing quality control and who were aged 18-40 in 1975.

### *S4S*

The S4S study recruited incoming students (n=15,067) at a large, urban, public university in the mid-Atlantic US, starting in 2011. All first-time freshmen aged >18 years were eligible to complete an online self-report survey, with follow-up surveys each subsequent spring in which they were enrolled at the university. Data collection was carried out using the secure REDCap system of electronic data capture tools (2). All participants provided informed consent, and the study was approved by the corresponding university Institutional Review Board. Full details on data collection have been previously published (8). The current study includes data from n=4,549 EUR participants from the first 5 cohorts whose DNA has been genotyped and passed quality control procedures.

## **Measures**

As ALSPAC, FinnTwin12, and S4S surveys were administered at structured times, some variability was observed in participants’ age at assessment for each wave. To prevent small cell counts and unreliable model estimates, we generally collapsed these and assigned all individuals to the most common age group within wave (e.g., all S4S participants were coded as age 18 at the first survey wave, while 6% of participants were actually aged 19 and 0.5% older than 19). When sample sizes per age were insufficient, we included multiple age bins (e.g., age ranges of 34-40 in the last wave of FinnTwin12 were recoded as 34-36=35 and 37-40=38). In AddHealth and COGA, we truncated the age range at 32, given the small number of observations outside this range. Based on the low prevalence of drinking initiation prior to adolescence and the ages/waves at which different cohorts had data available, we treated age 14 as the baseline for statistical models and excluded any data collected before this age. A timeline of the assessments for each cohort can be found in **Figure 1** and **Supplementary Table S1**.

### *CON*

Consumption frequency was measured using item #1 of the Alcohol Use Disorders Identification Test (AUDIT; 9) questionnaire (“How often do you have a drink containing alcohol?”) or similarly worded items, recording the number of typical drinking days in categories from “never” to “daily”. These categories were recoded into pseudo-continuous number of days per month, using the median value for categories with a range of days (i.e., 3 days for the category “2-4 times per month”) or the most conservative value when the range was non-specific (i.e., 16 days for the category “4 or more times a week”). This recoding allowed for a linear prediction of the variable on an interpretable quantitative scale that could be harmonized across cohorts.

### *HED*

Intoxication or HED frequency was measured using item #3 of the AUDIT questionnaire (“How often do you have six or more drinks on one occasion?”) or similarly worded items, recording the number of typical heavy drinking/intoxication days in categories from “never” to “daily”. As for CON, these categories were recoded into pseudo-continuous number of days per month. In FinnTwin12, the HED item was phrased as “How often do you drink so that you get at least slightly intoxicated?” (ages 14 and 17-18), “How often do you drink so that you get really drunk?” (age 21-26), and AUDIT item #3 (age 34-40). In COGA, the HED item was phrased as “How often did you have five or more drinks in 24 hours during the last 12 months?” and recoded to days per month. In S4S, HED was measured in two ways: 1) a sex-specific question “How often do you have [five/four] or more drinks in a single sitting (considered about a 2 hour period)?” for males/females, or 2) the values from the CON measure, above, if participants’ reported typical drinking quantity was >=5 drinks per day (otherwise HED frequency=0). The former measure was only available for a subset of waves; we took the maximum value when both measures were available.

### *Other AUB measures*

AUBs were measured somewhat differently in the FTC sample. However, because this is the only cohort with data collected into older adulthood (up to age 67), it provides important additional information about the trajectory of genetic risk later in life. We therefore include data from four AUBs measured in this sample. Frequency of alcohol use (“How often do you drink alcohol?”) was measured for beer, wine and spirits separately and the maximum value across beverage types within wave was taken (Freq) and recoded into a pseudo-continuous count of days per month. gEtOH was a composite measure of grams of ethanol consumption per month, x ↦ log(x+1) transformed, as previously described (10). Heavy was a dichotomous indicator of whether participants drink “more than five bottles of beer or more than a bottle of wine or more than half a bottle of hard liquor” on the same occasion, at least once a month. Finally, PassOut was a pseudo-continuous measure of the number of days in the past year that participants passed out due to alcohol consumption. Measures were binned in 2-year age intervals to stabilize estimation, and the minimum baseline age (intercept) was 18 for Freq, gEtOH, and Heavy, and 24 for PassOut, since this was assessed in the 1981 and later questionnaires but not the 1975 questionnaire.

## References

1. Harris KM, Halpern CT, Whitsel EA, Hussey JM, Killeya-Jones LA, Tabor J, et al. Cohort Profile: The National Longitudinal Study of Adolescent to Adult Health (Add Health). Int J Epidemiol. 2019;48(5):1415-k.

2. Harris PA, Taylor R, Thielke R, Payne J, Gonzalez N, Conde JG. Research electronic data capture (REDCap)--a metadata-driven methodology and workflow process for providing translational research informatics support. J Biomed Inform. 2009;42(2):377-81.

3. Boyd A, Golding J, Macleod J, Lawlor DA, Fraser A, Henderson J, et al. Cohort Profile: the 'children of the 90s'--the index offspring of the Avon Longitudinal Study of Parents and Children. Int J Epidemiol. 2013;42(1):111-27.

4. Fraser A, Macdonald-Wallis C, Tilling K, Boyd A, Golding J, Davey Smith G, et al. Cohort Profile: the Avon Longitudinal Study of Parents and Children: ALSPAC mothers cohort. Int J Epidemiol. 2013;42(1):97-110.

5. Begleiter H, Reich T. The Collaborative Study on the Genetics of Alcoholism. Alcohol Health & Research World. 1995;19(3):228.

6. Rose RJ, Salvatore JE, Aaltonen S, Barr PB, Bogl LH, Byers HA, et al. FinnTwin12 Cohort: An Updated Review. Twin Res Hum Genet. 2019;22(5):302-11.

7. Kaprio J, Bollepalli S, Buchwald J, Iso-Markku P, Korhonen T, Kovanen V, et al. The Older Finnish Twin Cohort - 45 Years of Follow-up. Twin Res Hum Genet. 2019;22(4):240-54.

8. Dick DM, Nasim A, Edwards AC, Salvatore JE, Cho SB, Adkins A, et al. Spit for Science: launching a longitudinal study of genetic and environmental influences on substance use and emotional health at a large US university. Front Genet. 2014;5:47.

9. Bohn MJ, Babor TF, Kranzler HR. The Alcohol Use Disorders Identification Test (AUDIT): validation of a screening instrument for use in medical settings. J Stud Alcohol. 1995;56(4):423-32.

10. Drouard G, Silventoinen K, Latvala A, Kaprio J. Genetic and Environmental Factors Underlying Parallel Changes in Body Mass Index and Alcohol Consumption: A 36-Year Longitudinal Study of Adult Twins. Obes Facts. 2023;16(3):224-36.

## Appendices

**Appendix 1. Glossary of abbreviations**

| AddHealth | the National Longitudinal Study of Adolescent to Adult Health cohort |
| --- | --- |
| ALSPAC | the Avon Longitudinal Study of Parents and Children cohort |
| AUB | alcohol use behavior |
| AUD | alcohol use disorder |
| AUDIT | alcohol use disorder identification test |
| COGA | the Collaborative Studies on the Genetics of Alcoholism cohort |
| CON | a measure of typical frequency of alcohol consumption |
| EUR | individuals with genetic ancestry similar to European reference panels |
| EXT | externalizing behavior |
| FinnTwin12 | the FinnTwin12 birth cohort of Finnish twins |
| Freq | a measure of frequency of alcohol use (days per month) |
| FTC | the older Finnish Twin birth cohort |
| gEtOH | a measure of grams of ethanol consumption (per month) |
| GWAS | genome-wide association study |
| Heavy | a measure of monthly heavy drinking (yes/no) |
| HED | a measure of typical frequency of heavy episodic drinking |
| *I^2^* | an estimate of effect heterogeneity between cohorts in a meta-analysis |
| IS(Q) model | LGC model with intercept and slope (and quadratic) latent growth factors |
| LGC | latent growth curve model |
| PassOut | a measure of frequency of passing out from alcohol (days per year) |
| PGS | polygenic score |
| S4S | the Spit for Science cohort |

# **Supplementary Tables**

**Supplementary Table S1. Descriptive statistics of alcohol use behaviors measured in five longitudinal cohorts.**

|  | AddHealth | | | ALSPAC | | | COGA | | | FinnTwin12 | | | S4S | | |
| --- | --- | --- | --- | --- | --- | --- | --- | --- | --- | --- | --- | --- | --- | --- | --- |
| Age | N | CON M (SD) | HED M (SD) | N | CON M (SD) | HED M (SD) | N | CON M (SD) | HED M (SD) | N | CON M (SD) | HED M (SD) | N | CON M (SD) | HED M (SD) |
| 14 | 1208 | 0.74 (2.95) | 0.54 (3.29) | x | x | x | 745 | 0.12 (0.80) | 0.05 (0.56) | 1171 | 0.45 (0.84) | 0.22 (0.53) | x | x | x |
| 15 | 1575 | 1.12 (3.36) | 0.84 (3.47) | x | x | x | 376 | 0.51 (1.87) | 0.30 (1.52) | x | x | x | x | x | x |
| 16 | 1672 | 1.5 (3.92) | 1.17 (3.94) | 3415 | 3.42 (3.48) | 0.99 (1.41) | 557 | 0.89 (2.60) | 0.65 (2.32) | x | x | x | x | x | x |
| 17 | 1734 | 1.88 (4.24) | 1.35 (3.95) | 2919 | 4.43 (3.97) | 1.10 (1.48) | 496 | 1.69 (3.45) | 1.11 (2.91) | 391 | 1.96 (2.11) | 0.91 (1.30) | x | x | x |
| 18 | 1434 | 2.39 (4.56) | 1.76 (4.28) | 2327 | 6.05 (4.63) | 1.56 (1.86) | 667 | 2.35 (4.65) | 1.52 (3.64) | 726 | 2.11 (2.18) | 0.90 (1.30) | 3770 | 2.77 (3.82) | 1.39 (3.32) |
| 19 | 881 | 3.22 (5.63) | 2.02 (4.44) | x | x | x | 559 | 3.67 (5.70) | 2.33 (4.55) | x | x | x | 3335 | 3.45 (3.98) | 2.00 (3.62) |
| 20 | 698 | 3.87 (6.08) | 2.21 (4.44) | 2934 | 5.68 (4.56) | 1.85 (2.04) | 649 | 4.25 (5.90) | 2.74 (5.09) | x | x | x | 2049 | 3.74 (4.06) | 2.54 (3.63) |
| 21 | 797 | 4.39 (6.04) | 2.04 (3.99) | x | x | x | 656 | 5.53 (6.35) | 3.09 (4.95) | x | x | x | 1342 | 5.17 (4.70) | 2.99 (4.04) |
| 22 | 769 | 4.37 (6.19) | 1.96 (4.57) | 2733 | 5.26 (4.44) | 1.44 (1.79) | 592 | 6.40 (6.91) | 3.45 (5.44) | 748 | 3.76 (3.75) | 1.43 (1.64) | 1060 | 5.89 (4.89) | 3.15 (4.11) |
| 23 | 769 | 4.53 (6.28) | 2.23 (4.79) | x | x | x | 556 | 6.41 (6.91) | 3.15 (5.24) | 465 | 4.03 (3.98) | 1.45 (1.67) | x | x | x |
| 24 | 662 | 4.31 (6.57) | 1.90 (4.58) | 2764 | 5.53 (4.56) | 1.39 (1.75) | 488 | 6.23 (6.93) | 2.85 (4.89) | x | x | x | x | x | x |
| 25 | 444 | 3.86 (5.69) | 1.64 (3.93) | x | x | x | 429 | 6.36 (6.68) | 2.44 (4.42) | x | x | x | x | x | x |
| 26 | 671 | 4.57 (6.44) | 1.74 (3.78) | x | x | x | 364 | 6.28 (7.36) | 2.33 (4.47) | x | x | x | x | x | x |
| 27 | 776 | 4.85 (6.86) | 2.02 (4.59) | x | x | x | 336 | 6.41 (7.22) | 2.26 (4.72) | x | x | x | x | x | x |
| 28 | 963 | 4.21 (6.48) | 1.61 (4.12) | 3010 | 5.2 (4.94) | 1.32 (2.08) | 261 | 6.61 (7.82) | 2.20 (4.64) | x | x | x | x | x | x |
| 29 | 873 | 4.14 (6.10) | 1.62 (3.91) | x | x | x | 232 | 7.13 (7.57) | 1.75 (3.63) | x | x | x | x | x | x |
| 30 | 931 | 4.30 (6.69) | 1.92 (4.9) | x | x | x | 191 | 6.27 (7.45) | 1.54 (3.61) | x | x | x | x | x | x |
| 31 | 577 | 4.56 (7.29) | 1.88 (4.92) | x | x | x | 140 | 6.45 (7.44) | 2.07 (4.62) | x | x | x | x | x | x |
| 32 | 70 | 4.19 (7.46) | 2.14 (5.43) | x | x | x | 190 | 5.86 (6.97) | 1.44 (3.59) | x | x | x | x | x | x |
| 33 | x | x | x | x | x | x | x | x | x | x | x | x | x | x | x |
| 34 | x | x | x | x | x | x | x | x | x | x | x | x | x | x | x |
| 35 | x | x | x | x | x | x | x | x | x | 269 | 3.16 (3.28) | 0.89 (1.60) | x | x | x |
| 36 | x | x | x | x | x | x | x | x | x | x | x | x | x | x | x |
| 38 | x | x | x | x | x | x | x | x | x | 334 | 3.13 (3.46) | 0.92 (1.55) | x | x | x |

*Note: CON=typical consumption frequency; HED=heavy episodic drinking frequency; x=no assessment available for this age/measure.*

**Supplementary Table S2. Descriptive statistics of alcohol use behaviors measured in the FTC cohort.**

| Age | N | Freq M (SD) | gEtOH M (SD) | Heavy M (SD) | PassOut M (SD) |
| --- | --- | --- | --- | --- | --- |
| 18 | 164 | 3.36 (2.89) | 4.09 (2.24) | 0.37 (0.48) | x |
| 20 | 519 | 3.93 (3.35) | 4.48 (1.99) | 0.35 (0.48) | x |
| 22 | 527 | 4.66 (3.79) | 4.98 (1.62) | 0.40 (0.49) | x |
| 24 | 606 | 4.71 (4.02) | 4.77 (1.83) | 0.36 (0.48) | 1.26 (1.73) |
| 26 | 959 | 4.15 (3.53) | 4.64 (1.79) | 0.33 (0.47) | 0.71 (1.25) |
| 28 | 977 | 4.44 (3.87) | 4.74 (1.73) | 0.31 (0.46) | 1.08 (1.76) |
| 30 | 968 | 4.49 (3.98) | 4.78 (1.72) | 0.31 (0.46) | 0.99 (1.52) |
| 32 | 1171 | 4.34 (4) | 4.6 (1.89) | 0.27 (0.44) | 0.94 (1.4) |
| 34 | 1319 | 4.54 (4.22) | 4.63 (1.87) | 0.26 (0.44) | 0.53 (1.28) |
| 36 | 1484 | 4.39 (3.95) | 4.60 (1.90) | 0.26 (0.44) | 0.45 (1.13) |
| 38 | 1640 | 4.42 (4.15) | 4.55 (1.95) | 0.26 (0.44) | 0.43 (1.14) |
| 40 | 1529 | 4.47 (4.24) | 4.52 (1.98) | 0.24 (0.43) | 0.48 (1.26) |
| 42 | 894 | 4.36 (4.26) | 4.41 (2.06) | 0.24 (0.43) | 0.38 (1.08) |
| 44 | 959 | 4.38 (4.31) | 4.39 (2.09) | 0.24 (0.43) | 0.37 (1.04) |
| 46 | 948 | 4.46 (4.37) | 4.41 (2.12) | 0.25 (0.43) | 0.44 (1.20) |
| 48 | 604 | 4.78 (4.44) | 4.52 (2.06) | 0.23 (0.42) | 0.26 (1.03) |
| 50 | 435 | 4.79 (4.62) | 4.46 (2.01) | 0.20 (0.40) | 0.20 (0.76) |
| 52 | 525 | 4.49 (4.43) | 4.29 (2.22) | 0.21 (0.41) | 0.20 (0.79) |
| 54 | 504 | 4.29 (4.57) | 4.25 (2.22) | 0.21 (0.41) | 0.31 (1.04) |
| 56 | 673 | 4.78 (4.72) | 4.72 (1.98) | 0.29 (0.45) | 0.47 (1.28) |
| 58 | 443 | x | 4.87 (1.86) | 0.33 (0.47) | 0.93 (1.40) |
| 60 | 397 | x | 4.94 (1.74) | 0.29 (0.46) | 0.68 (1.25) |
| 62 | 381 | x | 5.03 (1.73) | 0.28 (0.45) | 1.05 (1.65) |
| 64 | 360 | x | 5.01 (1.74) | 0.24 (0.43) | 0.76 (1.36) |
| 66 | 392 | x | 4.75 (1.85) | 0.21 (0.41) | 1.04 (1.64) |
| 67 | 76 | x | 5.26 (1.35) | 0.21 (0.41) | 0.75 (1.75) |

*Note: Freq=max frequency of consumption across different alcoholic beverage types; gEtOH=grams of ethanol consumption per month; Heavy=dichotomous measure of heavy drinking >1 time per month; PassOut=frequency of passing out from alcohol consumption in the past year; x=no assessment available for this age/measure.*

**Supplementary Table S3. Secondary meta-analysis of the polygenic score (PGS) prediction of latent growth factors underlying trajectories of typical consumption (CON) and heavy episodic drinking (HED) frequencies.**

|  | PGS | Factor | Meta β | Meta P | 95% CI | *I^2^* |
| --- | --- | --- | --- | --- | --- | --- |
| CON | Problems | Int | 0.158 | 0.022 | 0.023, 0.293 | 0.00 |
|  | Problems | Slope | **0.335** | **1.17E-06** | **0.200, 0.470** | 0.00 |
|  | Problems | Quad | -0.185 | 0.007 | -0.320, -0.050 | 0.00 |
|  | BeerPref | Int | 0.025 | 0.720 | -0.110, 0.160 | 0.01 |
|  | BeerPref | Slope | -0.020 | 0.887 | -0.296, 0.256 | 73.89 |
|  | BeerPref | Quad | -0.058 | 0.640 | -0.301, 0.185 | 66.60 |
|  | Consumption | Int | 0.155 | 0.307 | -0.142, 0.452 | 77.34 |
|  | Consumption | Slope | 0.361 | 0.002 | 0.137, 0.584 | 60.75 |
|  | Consumption | Quad | -0.101 | 0.507 | -0.398, 0.197 | 77.42 |
|  | EXT | Int | **0.404** | **4.65E-06** | **0.231, 0.576** | 36.19 |
|  | EXT | Slope | 0.191 | 0.391 | -0.245, 0.627 | 89.35 |
|  | EXT | Quad | -0.247 | 0.158 | -0.590, 0.096 | 82.92 |
| HED | Problems | Int | **0.276** | **8.59E-04** | **0.114, 0.438** | 28.30 |
|  | Problems | Slope | 0.298 | 0.004 | 0.095, 0.502 | 53.16 |
|  | Problems | Quad | -0.157 | 0.135 | -0.362, 0.049 | 53.91 |
|  | BeerPref | Int | 0.186 | 0.097 | -0.033, 0.406 | 59.53 |
|  | BeerPref | Slope | 0.085 | 0.642 | -0.274, 0.444 | 84.38 |
|  | BeerPref | Quad | -0.040 | 0.811 | -0.370, 0.289 | 81.52 |
|  | Consumption | Int | 0.006 | 0.931 | -0.129, 0.141 | 0.00 |
|  | Consumption | Slope | 0.347 | 0.005 | 0.102, 0.591 | 66.93 |
|  | Consumption | Quad | -0.201 | 0.219 | -0.522, 0.120 | 80.56 |
|  | EXT | Int | 0.501 | 0.004 | 0.164, 0.838 | 82.28 |
|  | EXT | Slope | 0.279 | 0.199 | -0.147, 0.704 | 88.81 |
|  | EXT | Quad | -0.259 | 0.162 | -0.623, 0.104 | 84.78 |

*Note: The table shows an alternate specification of the meta-analysis from Table 3, in which the S4S cohort has been excluded and the per-cohort effects are weighted by the square root of their respective sample sizes. Effect size was based on the t-test statistic in each cohort for the linear regression coefficient of the effect of the PGS on latent growth factors (Int, Slope, and Quad). T-test statistics were converted back to a standardized beta value (equivalent to the correlation between the PGS and the latent growth factor) using the escalc() function of the R metafor package. Columns show the effect size meta-analyzed across cohorts (Meta β) and its associated P-value and 95% confidence interval (CI). The I^2^ statistic is a metric of between-cohort heterogeneity in the effect size. Additional abbreviations are found in the glossary (Appendix 1) *significant after Bonferroni correction for 56 PGS-outcome pairs, p < .0009*

# Supplementary Figures

**Supplementary Figure S1. Forest plots of polygenic score (PGS) prediction of mean levels of typical consumption (CON) and heavy episodic drinking (HED) frequencies, meta-analyzed across five longitudinal cohorts.**

**Supplementary Figure S2. Forest plots of polygenic score (PGS) prediction of latent growth factors underlying trajectories of typical consumption (CON) frequency, meta-analyzed across five longitudinal cohorts.**

**Supplementary Figure S3. Forest plots of polygenic score (PGS) prediction of latent growth factors underlying trajectories of heavy episodic drinking (HED) frequency, meta-analyzed across five longitudinal cohorts.**
